# Post-operative shoulder imbalance in adolescent idiopathic scoliosis: a study of clinical photographs

**DOI:** 10.1186/s13013-015-0055-6

**Published:** 2015-11-17

**Authors:** K. Venugopal Menon, Haroon M. Pillay, Anbuselvam M, Naveen Tahasildar, Renjit Kumar J

**Affiliations:** Orthopaedics, Khoula Hospital, Mina al Fahal, Muscat, Sultanate of Oman; Department of Neurosurgery, RIPAS Hospital, Bandar Seri Begawan, Brunei Darussalam; Sparsh Hospital, Bangalore, India; Amrita Institute of Medical Sciences, Cochin, Kerala India

**Keywords:** Adolescent Idiopathic Scoliosis, Shoulder balance, Clinical photograph, Torso symmetry, Trunk balance, Neck symmetry

## Abstract

**Study design:**

Retrospective observational study.

**Objective:**

To assess what features determine post-operative shoulder asymmetry in Adolescent Idiopathic Scoliosis (AIS).

**Summary of background data:**

Shoulder balance is one of the major determinants of the cosmetic outcomes of AIS surgery. Yet, other than level of the shoulders we are not clear what parameters are to be measured to assess torso symmetry. This study looks at the various features that might affect the appearance of the shoulder region.

**Methods:**

The records of 157 operated cases of AIS were retrospectively reviewed. Eight patients with documented post-operative shoulder asymmetry and were dissatisfied with their cosmetic outcomes were selected for the study. Their clinical photographs alone were studied. Three regions- the base of the neck, the shoulder and upper arm region- were analysed separately. Four measures each for the neck and shoulder and two for the arms were documented. No statistical tools were employed since the numbers were quite small but consensus was obtained between two Consultant Orthopaedic surgeons regarding the cosmetic impact of each parameter.

**Results:**

The neck and the shoulder appeared independent determinants of cosmesis of the proximal trunk. The base of neck symmetry seemed to be dependent on four features viz. centralization of the neck, neck tilt, trapezius angle and base of neck angle. The appearance of the shoulder itself depended on its level, axillary fold level, scapular level and the scapular prominence. The upper arm parameters appeared less critical in determining the cosmetic impact.

**Conclusions:**

Proximal trunk symmetry in AIS depends on the symmetry of the base of the neck and shoulder regions. The level of the shoulders, axillary folds along with the base of neck angle, Trapezius angle appear to be key determinants of symmetry.

## Background

**Fig. 1:**
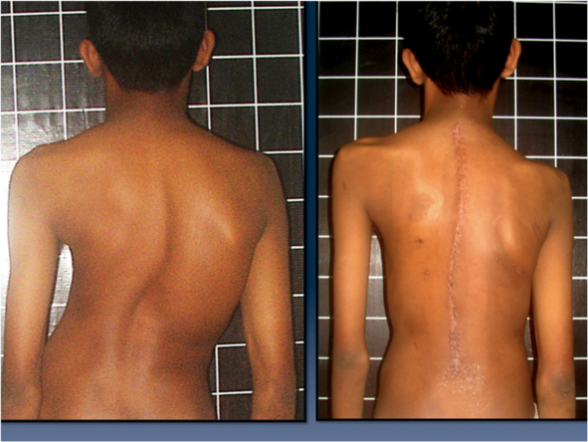
Pre and post-operative images of a patient of AIS. Note that despite the poor trunk alignment in the pre-op state the shoulders are balanced and after surgery though the trunk alignment has improved the shoulder has become unbalanced. In this patient the base of the neck features appear relatively preserved compared to the shoulder level

The overwhelming impact of evidence based medical practice is manifest by the need to describe and measure clinical parameters in detail-even qualitative data like satisfaction or cosmetic appearance needs to be defined and measured with precision [1]. In Adolescent Idiopathic Scoliosis (AIS) trunk symmetry, sagittal and coronal plane balance and shoulder balance are the features that are accepted as essential cosmetic considerations [2]. While there is uniform agreement that shoulder balance is of paramount importance, (Fig. 1) there is little consensus on what constitutes optimum shoulder symmetry in AIS patients. Traditionally the shoulder levels were measured by one of many methods radiologically [2–4]. Numerous radiological parameters have been described and their relative value in measuring shoulder balance compared including T1 vertebral tilt, Clavicle angle, Coracoid process height difference, Trapezius length, First Rib –Clavicle height, Clavicle rib cage intersection difference, First Rib angle, Clavicle tilt angle difference, Radiographic shoulder height etc. [5–10] Unfortunately it has been established that clinical shoulder balance or the absence of it does not always correlate well with radiological imbalance [2, 11, 12]. Winter in 1989 suggested that shoulder elevation, trapezial fullness and left thoracic rib prominence might be the clinical features of shoulder asymmetry seen in double thoracic curves [13]. Qui and co workers [11] were perhaps the first to point out the significance of clinical features of the proximal trunk imbalance and how to measure them on digital photographs. They have used pre-operative clinical photographs to determine areas of symmetry between the sides of the torso. Based on clinical photographs taken from posterior aspect, these authors also suggested that radiological balance does not always coincide with clinical symmetry of the shoulder. The research question then is ***what precise features are responsible for the unsightly appearance of the shoulder region in operated cases of AIS with shoulder imbalance***?

Cosmetic disfigurement in AIS is a major concern for patients and their parents [14] and it can be studied under several domains. The three most important ones that have been documented well are radiological measures of spine and trunk alignment (Cobb angle, Apical vertebral translation, spinal balance etc.), patient or parent perception of trunk appearance and body image, (Walter Reed Visual Analogue Scale-WRVAS, Spinal Assessment Questionnaire-SAQ etc.) and objective assessment of trunk symmetry on clinical photography or surface topography [15, 16]. These domains may be summarized as ***Radiological***, ***Patient Satisfaction*** and ***Clinical domains***. The idea of objective measurement of a subjective perception- cosmetic disfigurement- is not novel. Moire’s surface topography, ISIS scan, Quantec, Jenoptik Formetric, Raster photography etc. are but some of these [17–21]. Several authors have tried to introduce unprejudiced observational criteria into describing deformity [17, 20]. Clinical photography and videography have also been used for this purpose. The Trunk Aesthetic Clinical Evaluation is one such tool that uses clinical photographs for measuring cosmetic dysfunction where in addition to shoulder and scapular dissymmetry the waist and thoracic cage are taken into consideration [22]. Newer devices have also been described like the ISIS_2_. While these tools are typically used to determine the degree of disfigurement and its correction, specific measures of what constitutes right vs. left asymmetry of the torso, has not been clearly addressed.

This paper is an attempt to unveil what elements might be responsible for torso symmetry and therefore asymmetry after AIS surgery; hopefully this knowledge will help us better measure it and prevent or treat it.

## Materials and methods

The records of 157 operated cases of Adolescent Idiopathic scoliosis cases from two centers in Asia were reviewed retrospectively for this study. All of them had pre and post-operative clinical photographs that were available for review. There were 131 females and 26 males in the group and their mean age was 14.55 years. In the post operative follow-up records it was documented that eight patients had significant shoulder imbalance and were unhappy about their appearance. This included patients who were subjectively concerned about the appearance of their shoulders as well as cases where the clinician had recorded objective shoulder asymmetry (see Table 1). Four of them were boys and four were girls with a mean age of 15.06 years and an SD of 1.266. The clinical photographs of these eight cases formed the core materials for this study. All the post-operative photographs were taken at the 3 months review after surgery. Only the post-operative photographs were analysed for their cosmetic impact. In both centers clinical photographs were taken with a point and shoot digital camera at fixed distance of two meters. The camera was fixed at the level of the scapulae and focused at the base of the inter-scapular region*.* The patient stood relaxed against a blank wall or a grid (which was the standard practice in one of the institutions.) The footprints were marked on the floor for maximum reproducibility. The arms were held relaxed at the sides. Appropriate dress with tied up hair allowed maximum visibility of the neck and upper trunk without violating the patient’s cultural sensitivities (one patient with significant shoulder asymmetry but did not have an optimum post operative photograph is seen in Fig. 5). Only one standing image of the back view of the trunk was utilized for the purpose of this study.

**Table 1:** The eight cases and the various measures recorded are depicted. Angles are measured in degrees and lengths are expressed as percentage of the total length. Major aesthetic disfigurements are highlighted in pink

Sl No.	Name	Age	Gender	Neck tilt Le	Neck base Le	Trap Le	Neck center diff	Sh level	Ax level	Scap level	Scap prom	Arm width diff	Arm drop Le	clinical impression	Who noticed
1	MIZ	16	M	0	**12**	**12**	0.044	**6**	2	na	na	0	10	neck, sho	Dr
2	JT	15.5	M	2	**18**	**10**	4.40 %	3	1	2	yes	7.70 %	8	Neck	Pt/Dr
3	A	14	M	5	7	5	3.64 %	**10**	4	**15**	yes	18.50 %	0	Shoulder	Pt/Dr
4	NMMJ	14.5	F	na	na	na	na	**6**	3	0	0	9.10 %	10	shoulder	Pt/Dr
5	RH	14.5	M	3	6	**9**	4.76 %	5	4	0	0	0	2	Neck	Dr
6	FR	13.5	F	na	na	na	na	**7**	5	1	Yes	25 %	0	shoulder	Pt/Dr
7	AJ	17.5	F	0	7	8	0.05	**8**	5	6	0	21.21 %	1	shoulder	Pt/Dr
8	SS	15	F	2	**9**	4	0	4	3	**9**	yes	6.67 %	0	neck, sho	Pt/Dr
Mean		15.063													
SD		1.27													

Legends to Table:1

Column 5: Neck Tilt Angle

Column 6: Neck Base Angle

Column 7: Trapezius angle difference

Column 8: Neck Center difference

Column 9: Shoulder level

Column 10: Axillary level

Column 11: Scapular level

Column 12: Scapular Prominence

Column 13: Arm Width Difference

Column 14: Arm drop Angle

Column 15: depicts the 2 observer’s impression of the clinical photograph

The bold faced text suggests the measures for clinically significant disfigurement as assessed bycolumnn 15 of the table. However please note that these are not statistically tested

**Fig. 2:**
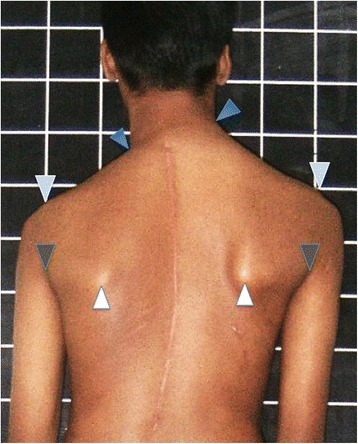
Post operative shoulder imbalance demonstrating the marking of the neck inflexion points (*Dark Blue arrows*), points of the shoulder (acromion) (*Light Blue arrows*), axillary folds (*Grey arrows*), and scapular prominence at its inferior poles (*White arrows*). In this case the base of the neck appears significantly tilted compared to the shoulder level itself

At one of the two institutions the Hospital information system had the capability to make angular, linear and area measurements on images and the measures were done digitally. At the other establishment the digital photographs were downloaded on to either a PowerPoint or Sketchbook Express page and the lower half of the body cropped out. Printouts were obtained and on these paired points were marked on the tips of the acromion, axillary folds, inferior poles of the scapulae, neck to Trapezius junction (inflexion point), and the center of the neck about the C7 spinous process (Fig. 2) The following lines and angles were measured on all the eight images manually-

**Fig. 3:**
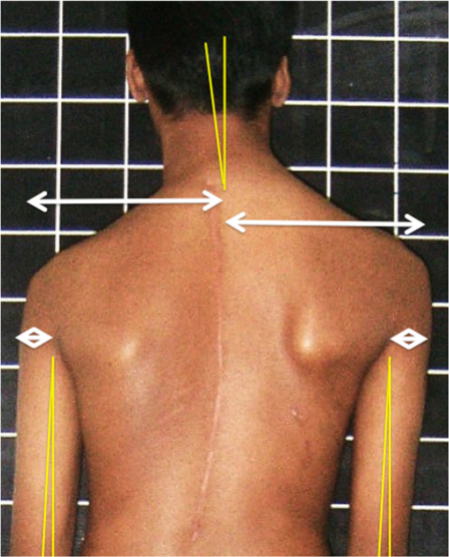
Illustrates the technique of measuring the axis of the neck in relation to the vertical axis and the centralization of the neck on the shoulders; additionally the width of the arm at the axillary fold level and the angle between the arm and vertical axis are marked out

**Fig. 4:**
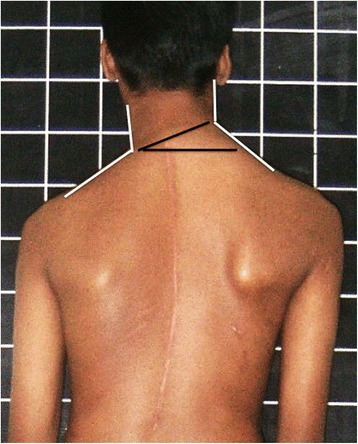
The Neck- Trapezius angle (*white lines*) is marked demonstrating a clear difference between the sides as well as the angle of the base of the neck (*black lines*) from the horizontal plane. Please note that the neck base appears significantly more tilted than the shoulders and axillary folds in this patient
- Base of the neck line and angle with the horizontal. Fig. 2 illustrates how the inflexion angle of the neck is marked and Fig. 4 shows how the angle is measured.
- Neck- Trapezius angle difference between the right and left. The angle at the inflexion point of the neck-Trapezius junction is measured and the difference documented (see Fig. 4).
- Neck inclination angle from the vertical. This is essentially the long axis of the neck against the vertical axis shown in Fig. 3. The axis of the neck is drawn by taking any two transverse diameters and joining their midpoints.
- Neck centralization- difference in the distance between the outer points of the shoulders to the center of the neck at C7 (expressed as percentage of the total distance between the shoulders). See white arrows in Fig. 3.
- Arm width difference- difference between arm width at the axillary fold level (expressed as percentage of the total arm width). Fig. 3.
- Arm drop angle- difference in angle between upper arm and vertical axis on either side (abduction is recorded as a positive value and adduction as a negative one) Fig. 3.

- Inter acromial line and angle with the horizontal. The line joining the points of the acromion were marked (light blue arrows in Fig. 2) and the angle with the horizontal measured.
- Inter axillary line and angle with the horizontal. The axillary folds were marked (grey arrows in Fig. 2) and the angle measured between the connecting line and the horizontal.
- Inferior poles of the scapulae connecting line and angle with the horizontal. White arrows in Fig. 2. illustrates the marking of these points.
- Differential prominence of the inferior scapular poles (documented as yes or No). See Figs. 2, 3, 4.

The final values from this series are presented in a tabular form in Table 1. Two clinicians evaluated all the eight images and decided where the aesthetic problem lies- shoulder, neck base, or upper arm or a combination of these. No statistical tools were used- only a consensus between the observers was obtained and documented in Table 1. The measures corresponding to major disfigurement were then identified from the table.

Figure 1 depicts a patient of AIS with post-operative shoulder imbalance despite excellent correction of the trunk deformity. Figure 2 illustrates the various points, lines and angles that are measured for this assessment of the shoulder domain while Fig. 3 demonstrates the “Arm” features as well as the neck tilt and neck centralization concept. Figure 4 clearly documents the base of neck angle and the Trapezius angle difference that were used in this study.

## Results

The results of this study are summarized in Table 1. Six of the eight patients recorded here displayed dissatisfaction with the appearance of their shoulder region while two were not aware of major aesthetic problems but the surgeon who reviewed them during follow up documented visible anomaly. By consensus between the two evaluating surgeons four patients had predominantly shoulder imbalance, two each had neck base and combined neck and shoulder imbalance. The arm features did not appear (to the observers) to make a major impact in the cases studied though some of the cases did have significant measured disparity (Cases 1,4 in terms of arm drop angles and cases 3 and 7 with regard to the arm width difference). It was observed that each region is independently capable of creating a perception of asymmetry though often deformities in both regions co-exist. For example the child in Fig. 2 demonstrates reasonable shoulder alignment but grossly distorted neck features. Similarly the patient depicted in Fig. 1 shows predominantly shoulder level dissymmetry affecting the acromial, axillary and scapular lines. Incidentally, this case also has asymmetric Trapezial angles and eccentric placement of the neck on the shoulders aggravating the cosmetic disfigurement.

The major disfigurement in the neck appeared to be inclination of the neck from the vertical axis (Fig. 2) but this was only an apparent phenomenon due to the base of neck being inclined. Though no statistical tools were employed due to the small number of cases in this study, from Table 1 it appears that neck base angle over 9^0^ as well as Trapezius angle difference over 9^0^ seem to correlate with significant unsightliness. (The power of the study did not allow cutoff values for each measure to be determined based on the data presented.) The centrality of the neck was measured by the distance from the edge of the shoulder to the center of the neck on either side. This parameter was noted to be abnormal frequently (Table 1) though less aesthetically damaging than other features. The difference in Trapezius angles on either side appeared to contribute substantially to visualized deformity. This was often better seen in boys than in girls and in thin children.

**Fig. 5:**
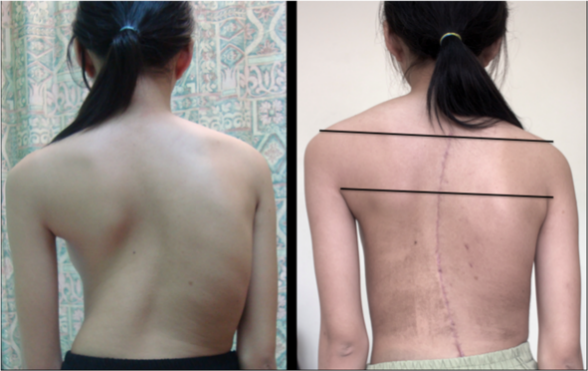
Figure depicts a patient with post-operative shoulder imbalance. However, the base of the neck is obscured by the hair though shoulder and axillary levels are clearly visible

In the shoulder itself major contributions to asymmetry were by the shoulder level and to a lesser extent by the axillary fold level and scapular prominence. A shoulder level angle of 6^0^ and a scapular angle of 9^0^ appeared to be the threshold for aesthetic impact though again not statistically validated due to small numbers. The level of the axillary fold, its shape and contribution to the body contour impacts the overall appearance though in this study it had a lesser impact than shoulder level. Prominent scapular poles were seen equally in patients with significant shoulder asymmetry as well as those without making the contribution of this feature less reliable than imagined. When assessing the axillary fold it was observed that the width of the upper arm seemed to be different on both sides suggesting that there might be an element of trunk rotation that affects the appearance of the torso as well (Fig. 5). The arm width and the long axis of the upper arm appeared to have a much lesser impact on appearance despite the measured difference between sides.

The influence of each component of the deformity on the overall unsightliness is variable in each given case. Moreover, in each patient there are combinations of elements that contribute significantly and others that matter a little less to the general disfigurement. Perhaps there are particularly unpleasant combinations- in this case series the numbers were too small to analyse all these factors. There is also a subjective element to the cosmetic influence of each component of the deformity; again in this study neutral observers were not employed for the assessment of impact of each feature.

## Discussion

Shoulder balance has long been recognized as a major contributor to the overall trunk appearance after scoliosis surgery. When a right thoracic curve with a high right shoulder was operatively corrected most often the shoulder balanced itself post operatively. The discovery of the double thoracic curve and the left shoulder elevation introduced confusion in the instrumentation strategy. A lot of emphasis was initially placed on the proximal thoracic curve and its role in determining the level of the shoulder. Most authors have reported that structural Proximal Thoracic (PT) curve would result in the left shoulder being elevated and nonstructural ones would lead to right shoulder elevation [23]. The criteria for structurality of curves varied between authors. As a natural consequence a lot of literature emerged on selecting Upper Instrumented Vertebra (UIV) to achieve balanced shoulders [8–10].

Simultaneously several authors started looking at the radiographic criteria to define shoulder balance [24]. Since it was soon established that the T1 vertebral tilt traditionally used to bench mark shoulder level was not a reliable indicator of clinical shoulder level, multiple other measures like Clavicle angle, Coracoid process height difference, Trapezius length, First Rib –Clavicle height, Clavicle rib cage intersection difference, First Rib angle, Clavicle tilt angle, Clavicle chest cage angle difference, First Rib Index etc. emerged [25, 26]. In recent years it has become increasingly apparent that radiological parameters of shoulder level do not always coincide with clinical levels and that the patients’ subjective perception of body image has little to do with the x-ray appearance [2]. Along with the SRS outcome tools a number of other patient satisfaction measures were developed. The Walter Reed Visual Assessment Scale was one of the earliest and most comprehensive [27]. It did estimate the patient’s perception of shoulder balance in two domains- shoulder levels and scapular prominence. Though there is no numerical description of the five grades in each domain and it looks at the posterior aspect of the body only, it was a major contributor to our understanding of shoulder symmetry in AIS. The authors later upgraded the questionnaire eliminating the scapular prominence and incorporating a lateral view instead (SAQ) [28, 29]. Bago and colleagues introduced the Trunk Appearance Perception Scale (TAPS) questionnaire and reported good inter-observer reliability using this system [30]. The shoulder, trunk and waist as seen from the back were objectively studied by Zaina et al. [22] to formulate the TRACE tool which has been found to be fairly reproducible by the authors. But the question remained- what makes the patient’s shoulder look unsightly? Is it just the shoulder level and scapular prominence or are there other factors to this cosmetic perception? The current study has addressed these questions to some measure.

The three domains of shoulder balance may be summarized as radiological, subjective perception (this includes parent’s input as well) and objective (clinical photography). The former two domains have been addressed elaborately as per the foregoing discussion. Qui et al. [11] made the first comprehensive study of clinical shoulder asymmetry using clinical photographs. These authors used pre-op photographs and described several tools that may help measure the various shoulder and base of neck elements accurately. However they did not describe the components of torso disfigurement that sometimes occurs after AIS surgery that is the main emphasis of the present study. The TRACE study also utilizes clinical photographs pictured from the back.

From the results of this study it is apparent that shoulder asymmetry has two major components- the base of the neck and the shoulder/scapula/axillary region. Each region has several elements that can individually or collectively affect the aesthetic appeal of that area. For the neck region the tilt of the neck from the vertical axis, the angle of the base of the neck, symmetry and level of trapezius angle and the centrality of the neck upon the shoulder are the chief elements of deformation. In the shoulder region the contributors are the level of the shoulders itself, level and shape of the axillary folds, and the scapular prominence-its size and level. Obviously various permutations and combinations of these are possible and only very large series of cases can represent every possible combination. Fortunately post-operative shoulder imbalance is rare and therefore only large multicenter studies can gather the data required. Radiological correlates to these individual elements has not been attempted in the current study but it would seem interesting to postulate that the neck features would depend more on the T1 tilt and the shoulder features on the ICL (inter coracoid line). The authors have established in a previous study that the T1 tilt and ICL are often independent of each other [31] and a second prospective study is under way with larger numbers involving pre-operative scoliosis patients addressing their shoulder balance.

The present study has important implications to clinical practice. When comparing pre and post-operative images clinicians can have a more objective tool to document the contrast in shoulder balance. It appears that the base of the neck and the shoulder (including axillary and scapular levels) features determine the aesthetic appeal of the upper trunk region. The upper arm dissymmetry has a lesser role in the body image. Each of these features can independently or collectively cause disfigurement ascribed as post-operative shoulder imbalance. The current understanding of shoulder symmetry may eventually help us prevent shoulder imbalance during AIS surgery.

This study has several limitations. The assessment of aesthetic appeal was performed by two physicians and only consensus was obtained; multiple non-medical judges and statistical tools were not employed. Only posterior views were evaluated for this study. Anterior and lateral images were not studied for socio-cultural reasons. All the photographs were done in the early post op period. It is certainly possible that many of the early shoulder decompensations spontaneously resolved over time though it is believed that this is less likely to occur than trunk imbalance. It is hypothesised that the base of neck features relate to the T1 tilt and the shoulder features to the ICL (inter coracoid line) but such a radiological correlation was not attempted in this study. The number of cases out of necessity was small and were all operated by one senior surgeon. Obviously larger, multicenter studies looking at different combinations of photographic features and their impact on shoulder cosmesis need to be conducted.

## Conclusions

Post operative shoulder imbalance consists of neck asymmetry measured by neck axis tilt, neck base obliquity, lack of neck centralization and Trapezius angle asymmetry and, shoulder dissymmetry measured by uneven shoulder levels, axillary fold unevenness, scapular level obliquity and unequal scapular prominence. Each of these two regions can independently affect aesthetic appeal and often exist in combination with other regional asymmetries. Measuring these parameters would objectively document post-operative shoulder imbalance after Scoliosis surgery.

## Abbreviations

AIS: adolescent Idiopathic Scoliosis
SAQ: spinal assessment questionnaire
TAPS: trunk appearance perception scale
TRACE: Trunk Aesthetic Clinical Evaluation
UIV: upper instrumented vertebra
WRVS: Walter Reed Visual Assessment Scale
